# Relationship of body mass index to percent body fat and waist circumference among schoolchildren in Japan - the influence of gender and obesity: a population-based cross-sectional study

**DOI:** 10.1186/1471-2458-10-493

**Published:** 2010-08-18

**Authors:** Hirotaka Ochiai, Takako Shirasawa, Rimei Nishimura, Aya Morimoto, Naoki Shimada, Tadahiro Ohtsu, Emiko Kujirai, Hiromi Hoshino, Naoko Tajima, Akatsuki Kokaze

**Affiliations:** 1 Department of Public Health, Showa University School of Medicine, Tokyo, Japan; 2 Division of Diabetes, Metabolism and Endocrinology, Department of Internal Medicine, Jikei University School of Medicine, Tokyo, Japan

## Abstract

**Background:**

Although the correlation coefficient between body mass index (BMI) and percent body fat (%BF) or waist circumference (WC) has been reported, studies conducted among population-based schoolchildren to date have been limited in Japan, where %BF and WC are not usually measured in annual health examinations at elementary schools or junior high schools. The aim of the present study was to investigate the relationship of BMI to %BF and WC and to examine the influence of gender and obesity on these relationships among Japanese schoolchildren.

**Methods:**

Subjects included 3,750 schoolchildren from the fourth and seventh grade in Ina-town, Saitama Prefecture, Japan between 2004 and 2008. Information about subject's age, sex, height, weight, %BF, and WC was collected from annual physical examinations. %BF was measured with a bipedal biometrical impedance analysis device. Obesity was defined by the following two criteria: the obese definition of the Centers for Disease Control and Prevention, and the definition of obesity for Japanese children. Pearson's correlation coefficients between BMI and %BF or WC were calculated separately for sex.

**Results:**

Among fourth graders, the correlation coefficients between BMI and %BF were 0.74 for boys and 0.97 for girls, whereas those between BMI and WC were 0.94 for boys and 0.90 for girls. Similar results were observed in the analysis of seventh graders. The correlation coefficient between BMI and %BF varied by physique (obese or non-obese), with weaker correlations among the obese regardless of the definition of obesity; most correlation coefficients among obese boys were less than 0.5, whereas most correlations among obese girls were more than 0.7. On the other hand, the correlation coefficients between BMI and WC were more than 0.8 among boys and almost all coefficients were more than 0.7 among girls, regardless of physique.

**Conclusions:**

BMI was positively correlated with %BF and WC among Japanese schoolchildren. The correlations could be influenced by obesity as well as by gender. Accordingly, it is essential to consider gender and obesity when using BMI as a surrogate for %BF and WC for epidemiological use.

## Background

Obesity in childhood increases the risk of obesity in adulthood and is associated with cardiovascular disease (CVD) risk factors [1]. Therefore, childhood obesity should be closely monitored.

The ideal definition of child obesity, based on percent body fat (%BF), is impracticable for epidemiological use [2] because a special device, which is not always available, is needed to measure %BF. Although waist circumference (WC) is a well-known predictor of metabolic syndrome [3,4] and is associated with CVD risk factors among children [5], it is not usually measured in annual health examinations at elementary schools or junior high schools in Japan.

On the other hand, height and weight are commonly measured in annual health examinations among schoolchildren and, as a result, body mass index (BMI) can be calculated. If BMI showed a close correlation with %BF or WC, BMI could be useful as a surrogate for %BF and/or WC in childhood obesity or metabolic syndrome monitoring. Although the correlation coefficient between BMI and %BF or WC has been reported [3,6-12], studies conducted among population-based schoolchildren to date have been limited in Japan. Therefore, it is important to evaluate the relationship of BMI to %BF and WC among population-based samples in Japan.

The aim of this study was to investigate the relationship between BMI and %BF or WC among Japanese schoolchildren. We also examined the influence of gender and obesity on these relationships.

## Methods

This study was conducted as part of a pediatric health promotion program initiated in 1994 in Ina-town, Saitama Prefecture, Japan. In addition to annual national health checkups performed in accordance with the School Health Law, Ina-town has a unique health-promotion program. Of the fourth and seventh graders who had undergone regular health checkups, those who volunteered to take part in the program underwent blood and physical examinations. We have reported the results of this health promotion program previously [3,6,13-17].

### Study subjects

Subjects of this study included all schoolchildren from the fourth grade (aged 9 or 10 years) and seventh grade (aged 12 or 13 years) in Ina-town during 2004-2008. Informed consent to participate in the study was obtained from the parent or guardian of each child. This study was approved by the Medical Ethics Committee of Showa University School of Medicine.

### Information collection

The following information was obtained from each subject: age, sex, height, weight, %BF, and WC. These items were annually recorded in a health promotion program during 2004-2008. The measurements of height, weight, %BF, and WC were performed in the school's infirmary or in a designated room that protected privacy during the procedures. Subjects were asked to remove shoes and socks before measurement. Height was measured to the nearest 0.1 cm using a stadiometer, and body weight was measured with a scale to the nearest 0.1 kg. BMI was calculated as weight (kg) divided by height (m) squared. %BF was measured in the morning with a bipedal biometrical impedance analysis (BIA) device (Model TBF-102, Tanita, Tokyo, Japan) to the nearest 0.1%, over light clothing in a standing position. WC was measured in a standing position at the navel level while another examiner checked verticality from the side. Height, weight, %BF, and WC were measured one time for each subject.

### Definition of obesity

Obesity (not including overweight) was defined by using two criteria: the obese definition of the Centers for Disease Control and Prevention (CDC) [18], which is based on the weight status category for the BMI-for-age percentile (equal to or greater than the 95th percentile); and the definition of obesity in Japanese children by Asayama et al [19], which consists of both percentage of overweight (> 20% for childhood under 18 years old) and %BF (> 25% for boys; > 30% for girls younger than 11 years, and > 35% for girls with age of 11 years or older).

### Data analysis

Statistical analysis was performed among fourth and seventh graders separately to consider age differences. Unpaired t-tests or chi-squared tests were used to compare various characteristics between boys and girls. Pearson's correlation coefficients between BMI and %BF or WC were calculated. The correlation coefficient was applied separately for sex (boys or girls). A *P*-value of less than 0.05 was considered statistically significant. All statistical analyses were performed using Statistical Analysis System (SAS, version 9.1).

## Results

Of 3,792 subjects, 3,759 participated in this study (participation rate: 99.1%). Among 3,759 schoolchildren, 9 were excluded because of missing data. Thus, 3,750 children (1,932 boys and 1,818 girls) were analyzed.

Baseline characteristics of study subjects are shown in Table 1. There were 1,054 boys and 990 girls among the fourth-grade participants. The mean age was about 9.3 years among boys and 9.4 years among girls (*P *= 0.460). No statistical difference between boys and girls was observed regarding height. However, values for weight (31.6 vs. 30.6, *P*< 0.001), BMI (17.2 vs. 16.7, *P*< 0.001), %BF (19.2 vs. 16.9, *P*< 0.001), and WC (59.5 vs. 58.5, *P *= 0.002) were significantly greater among boys than girls. Among 1,706 seventh graders, 878 were boys and 828 were girls. There was no significant difference between boys and girls in age, whereas values for height (154.9 vs. 152.9, *P*< 0.001) and weight (45.4 vs. 44.0, *P *= 0.002) were significantly higher among boys than girls. There were no significant differences between boys and girls in BMI or WC; in contrast, %BF was significantly higher among girls than boys (20.8 vs. 16.4, *P*< 0.001).

**Table 1:** Baseline characteristics of study subjects

	Fourth graders (aged 9 or 10 years)			Seventh graders (aged 12 or 13 years)		
		
	Boys (n = 1,054)	Girls (n = 990)	P-value^a^	Boys (n = 878)	Girls (n = 828)	P-value^a^
Age (years)	9.3 (0.5)	9.4 (0.5)	0.460	12.3 (0.5)	12.3 (0.5)	0.858
Height (cm)	135.0 (5.8)	134.8 (6.4)	0.421	154.9 (8.1)	152.9 (6.0)	< 0.001
Weight (kg)	31.6 (6.5)	30.6 (6.2)	< 0.001	45.4 (10.4)	44.0 (8.0)	0.002
BMI (kg/m^2^)	17.2 (2.7)	16.7 (2.4)	< 0.001	18.8 (3.3)	18.7 (2.7)	0.900
%BF (%)	19.2 (5.6)	16.9 (5.4)	< 0.001	16.4 (5.9)	20.8 (5.9)	< 0.001
WC (cm)	59.5 (7.7)	58.5 (6.6)	0.002	65.8 (9.2)	65.3 (7.2)	0.233
Number of obesity^b^	87 (8.3)	31 (3.1)	< 0.001	53 (6.0)	18 (2.2)	< 0.001
Number of obesity^c^	79 (7.5)	29 (2.9)	< 0.001	51 (5.8)	18 (2.2)	< 0.001

Values are means (SD) or number (%)

BMI, body mass index; %BF, percent body fat; WC, waist circumference; SD, standard deviation

^a^unpaired t-test or chi-squared test

^b^Obesity is defined according to the obese definition of the Centers for Disease Control and Prevention

^c^Obesity is based on the definition of obesity in Japanese children

Table 2 shows the Pearson's correlation coefficients of BMI and %BF or WC for each gender by grade. Among fourth graders, the correlation coefficients between BMI and %BF were 0.74 for boys and 0.97 for girls, whereas those between BMI and WC were 0.94 for boys and 0.90 for girls. Similar results were observed in the analysis of seventh graders. The correlation coefficients between BMI and %BF or WC were 0.69 or 0.94 among boys and 0.96 or 0.88 among girls.

**Table 2:** Correlations of body mass index (BMI) and percent body fat (%BF) or waist circumference (WC) for each gender by grade

	Fourth graders		Seventh graders	
		
	Boys (n = 1,054)	Girls (n = 990)	Boys (n = 878)	Girls (n = 828)
%BF vs. BMI	0.74	0.97	0.69	0.96
WC vs. BMI	0.94	0.90	0.94	0.88

Fourth graders are aged 9 or 10 years

Seventh graders are aged 12 or 13 years

Values are Pearson's correlation coefficients

All values were statistically significant

Next, the analysis was limited to either boys or girls, and the correlation coefficient was calculated by obese or non-obese as defined by the CDC (Table 3). In the analysis of boys, the coefficients between BMI and %BF among fourth graders were 0.60 for the non-obese group and 0.45 for the obese group, while those among seventh graders were 0.54 for the non-obese and 0.20 (no statistical significance) for the obese. Therefore, the correlation coefficients between BMI and %BF were consistently weaker among the obese than the non-obese. Although the same tendency was observed in the correlation between BMI and WC, the correlation coefficients were more than 0.8 among both the non-obese (fourth graders: 0.87, seventh graders: 0.87) and the obese (0.86, 0.85). Analysis for girls showed relatively stronger correlation coefficients among the non-obese than among the obese. The coefficients between BMI and %BF among the non-obese or obese were 0.96 or 0.71-0.78, while those between BMI and WC were 0.85-0.86 or 0.72-0.79 among the non-obese or obese, respectively; the correlations between BMI and %BF or WC were more than 0.7 regardless of grade and physique (obese or non-obese)

**Table 3:** Correlations of body mass index (BMI) and percent body fat (%BF) or waist circumference (WC) by obesity or non-obesity according to the obese definition of Centers for Disease Control and Prevention

	Boys				Girls			
		
	Fourth graders		Seventh graders		Fourth graders		Seventh graders	
				
	Non-obesity (n = 967)	Obesity (n = 87)	Non-obesity (n = 825)	Obesity (n = 53)	Non-obesity (n = 959)	Obesity (n = 31)	Non-obesity (n = 810)	Obesity (n = 18)
%BF vs. BMI	0.60	0.45	0.54	0.20	0.96	0.71	0.96	0.78
WC vs. BMI	0.87	0.86	0.87	0.85	0.86	0.72	0.85	0.79

Fourth graders are aged 9 or 10 years

Seventh graders are aged 12 or 13 years

Values are Pearson's correlation coefficients

All values are statistically significant except the value of obese seventh-grade boys

Similar results were seen using the definition of obesity for Japanese children by Asayama et al (Table 4). Among boys, the correlation coefficients between %BF and BMI were less than 0.6, whereas those between WC and BMI were about 0.9. Moreover, the correlations of BMI and %BF were less than 0.6 for the obese, whereas those of BMI and WC were approximately 0.9 for the obese. On the other hand, the coefficients between BMI and %BF or WC among girls were at least 0.7 regardless of grade and physique.

**Table 4:** Correlations of body mass index (BMI) and percent body fat (%BF) or waist circumference (WC) by obesity or non-obesity according to the definition of obesity in Japanese children

	Boys				Girls			
		
	Fourth graders		Seventh graders		Fourth graders		Seventh graders	
				
	Non-obesity (n = 975)	Obesity (n = 97)	Non-obesity (n = 827)	Obesity (n = 51)	Non-obesity (n = 961)	Obesity (n = 29)	Non-obesity (n = 810)	Obesity (n = 18)
%BF vs. BMI	0.57	0.53	0.52	0.17	0.96	0.70	0.95	0.83
WC vs. BMI	0.89	0.90	0.89	0.89	0.87	0.76	0.85	0.78

Fourth graders are aged 9 or 10 years

Seventh graders are aged 12 or 13 years

Values are Pearson's correlation coefficients

All values are statistically significant except the value of the obese seventh-grade boys

## Discussion

### Baseline characteristics of study subjects

Mean height and weight in this study subjects (Table 1) were similar to those in general Japanese children aged 9-10 or 12-13 years, according to national statistics of Ministry of Education, Culture, Sports, Science and Technology [20]. Therefore, the differences between boys and girls were thought to be common among fourth graders and seventh graders in Japan. However, it could be essential to consider the sex differences in baseline characteristics when investigating the relationship between BMI and %BF or WC. Accordingly, we analyzed the data separately for sex and examined the relationship of BMI to %BF and WC.

### Gender differences

In the analysis of correlations of BMI and %BF or WC for each gender by grade (Table 2), the correlation coefficients between BMI and %BF or WC among boys were 0.69-0.74 or 0.94, whereas those were 0.96-0.97 or 0.88-0.90 among girls. Therefore, the correlations between BMI and %BF among girls were consistently stronger than those among boys, whereas those between BMI and WC among girls were relatively weaker than those among boys. These findings are consistent with previous studies [3,6,9,10,12]. Because a past study has reported the relationship between pubertal alternations in sex steroids, growth hormones, etc., and changes in body composition and fat distribution [21], it is important to consider gender to address the relationship between BMI and %BF or WC. Further study, however, is needed to elucidate the physiologic mechanisms of gender differences.

### The influence of obesity

The correlation coefficient between BMI and %BF varied by physique, with a weaker correlation among the obese, regardless of the definition of obesity. In addition, most correlation coefficients among obese boys were less than 0.5, whereas those were more than 0.7 among obese girls. Sexual dimorphism in human body composition emerges primarily during puberty, and the difference can be attributed to the action of sex hormones [22]; for example, testosterone is important for the increase in lean mass that occurs during puberty, especially in boys. Therefore, the influence of lean mass on the obese could be stronger in boys and the influence varies among individuals. As a result, the variability of %BF at a given BMI among girls could be smaller than that among boys. Concurrently, these findings may indicate that the sample size of obese children in this study might be too small compared with that of non-obese children, resulting in susceptibility to outliers. The results suggest that BMI is not utilizable as a predictor of %BF among the obese, especially in boys. Accordingly, it could be necessary to take physique into consideration when discussing the relationship between BMI and %BF.

The correlation coefficients between BMI and WC were more than 0.8 among boys and almost all coefficients among girls were more than 0.7, regardless of physique. A previous study in children showed that excess fat storage is initially subcutaneous rather than visceral [23]. Therefore, physique could have little effect on the correlation between WC, which is a useful surrogate for the visceral adipose tissue area [19], and BMI, which is the best predictor of visceral fat [24]. These findings indicate that BMI could be useful as a surrogate measurement of WC regardless of gender and physique.

### Limitations

There are a few limitations to this study. First, %BF was measured by BIA rather than dual-energy X-ray absorptiometry (DEXA), which is thought to be a reliable method for measurement of body composition [25]. Therefore, the accuracy of the present results is likely to be less than that of studies using DEXA. However, previous study reported that %BF showed a close correlation when measured by BIA and DEXA with the correlation coefficient being 0.90 [25]. Furthermore, our study results were not inconsistent with those of another study using DEXA [9]. These findings suggest that the results of this study are reasonable.

In addition, the correlation coefficient could be affected by outliers because Pearson's correlation coefficient was used in this study. However, similar results were observed even when Spearman's rank correlation coefficient was calculated (data not shown). Therefore, findings in this study would be not substantially influenced by outliers.

Finally, subjects in this study were from one town of Japan. Therefore, it might be difficult to apply the results of this study to general Japanese children or other races, as race differences in correlations between BMI and %BF have been shown [9].

## Conclusions

BMI was positively correlated with %BF and WC among Japanese schoolchildren. The correlations could be influenced by obesity as well as by gender. Accordingly, it is essential to consider gender and obesity when using BMI as a surrogate for %BF and WC for epidemiological use.

## Competing interests

The authors declare that they have no competing interests.

## Authors' contributions

HO, TS, and NS planned this study. RN and AM contributed to improve this study in a meaningful way. HO drafted this manuscript. TS, RN, and AM performed the data collection. TS was in charge of the supervision of the data collection. EK and HH supported the data collection. HO, NS, and TO contributed to the statistical analysis. NT and AK made substantial contributions to the conception of this study and the revision of the manuscript. All authors read and approved the final manuscript.

## Pre-publication history

The pre-publication history for this paper can be accessed here:

http://www.biomedcentral.com/1471-2458/10/493/prepub
